# Analysis of Integrin α_IIb_ Subunit Dynamics Reveals Long-Range Effects of Missense Mutations on Calf Domains

**DOI:** 10.3390/ijms23020858

**Published:** 2022-01-13

**Authors:** Sali Anies, Vincent Jallu, Julien Diharce, Tarun J. Narwani, Alexandre G. de Brevern

**Affiliations:** 1 INSERM, BIGR, Université de Paris and Université de la Réunion et Université des Antilles, F-75015 Paris, France; sali.anies@etu-univ-paris-diderot.fr (S.A.); julien.diharce@univ-paris-diderot.fr (J.D.); tjrnarwani@gmail.com (T.J.N.); 2 Centre National de Référence en Hémobiologie Périnatale (CNRHP), Site St Antoine, DMU Biologie et Génomique Médicales, AP-HP, Sorbonne Université, F-75012 Paris, France; vincent.jallu@aphp.fr

**Keywords:** secondary structure, sequence–structure relationship, structural alphabet, bleeding disorder, flexibility, molecular dynamics, Glanzmann thrombasthenia, blood group, human platelet antigens

## Abstract

Integrin α_IIb_β_3_, a glycoprotein complex expressed at the platelet surface, is involved in platelet aggregation and contributes to primary haemostasis. Several integrin α_IIb_β_3_ polymorphisms prevent the aggregation that causes haemorrhagic syndromes, such as Glanzmann thrombasthenia (GT). Access to 3D structure allows understanding the structural effects of polymorphisms related to GT. In a previous analysis using Molecular Dynamics (MD) simulations of α_IIb_ *Calf-1* domain structure, it was observed that GT associated with single amino acid variation affects distant loops, but not the mutated position. In this study, experiments are extended to *Calf-1, Thigh,* and *Calf-2* domains. Two loops in *Calf-2* are unstructured and therefore are modelled expertly using biophysical restraints. Surprisingly, MD revealed the presence of rigid zones in these loops. Detailed analysis with structural alphabet, the Proteins Blocks (PBs), allowed observing local changes in highly flexible regions. The variant P741R located at C-terminal of *Calf-1* revealed that the *Calf-2* presence did not affect the results obtained with isolated *Calf-1* domain. Simulations for *Calf-1* + *Calf-2*, and *Thigh* + *Calf-1* variant systems are designed to comprehend the impact of five single amino acid variations in these domains. Distant conformational changes are observed, thus highlighting the potential role of allostery in the structural basis of GT.

## 1. Introduction

Integrins are a large protein family composed of heterodimeric receptors composed of α and β subunits [1]. A total of 24 different combinations of α and β subunits are found in human cells. The human integrin α_IIb_β_3_ is an essential complex implicated in fibrinogen-dependent platelet aggregation and thrombus formation, thus maintaining primary hemostasis [2].

Each subunit of the α_IIb_β_3_ structure can be defined in 3 regions with the largest one, i.e., the extracellular ectodomain (959 and 693 residues in α and β subunits respectively), being a single spanning transmembrane region and a small C-terminus cytoplasmic region. The α_IIb_β_3_ structure is found in an inactive conformation of α_IIb_β_3_, i.e., its closed structure having been successfully crystallized [3]. It is hypothesized that the presence of fibrinogen stimulates the pathways resulting in the opening of the α_IIb_β_3_ structure. The opening results in a separation of the two subunits, maintaining an interface at one position that is critical for the binding of fibrinogen [4] (see Figure 1 of [5] for more details). The association of the transmembrane helices with α_IIb_ and β_3_ subunits plays a critical role in maintaining the inactive state [6].

As the human integrin α_IIb_β_3_ is a platelet surface fibrinogen receptor, it is responsible for platelet aggregation and, therefore, its deficiency/dysfunction is linked to life-threatening bleeding disorders. The first one is a rare autosomal recessive genetic disease associated with impaired α_IIb_β_3_ expression and/or function, namely Glanzmann thrombasthenia (GT). Bleeding occurs as the platelet aggregation and thrombus formation fails in the absence or functional impairment of integrin α_IIb_β_3_. This rare disease is associated to more than 350 different genetic variations, and many lead to amino acid substitutions (polymorphism) in every region of the two subunits. ClinVar lists 129 different ones for β_3_ (https://clinvarminer.genetics.utah.edu/variants-by-gene/ITGB3/condition/Glanzmann%20thrombasthenia, accessed on 16 November 2021) and 236 for α_IIb_ (https://clinvarminer.genetics.utah.edu/variants-by-gene/ITGA2B/condition/Glanzmann%20thrombasthenia, accessed on 16 November 2021). These variants have different consequences. Some prevents the expression of integrin α_IIb_β_3_ on the surface of platelets whereas others limit its expression, resulting in a reduced number of integrins on the platelet surface. The latter limits the efficiency of signal transduction [7,8], resulting in weak interactions for thrombus formation. It must also be noted that some rare gain-of-function mutations affecting the structure of α_IIb_ or β_3_ can give rise to macrothrombocytopenia associated with autosomal dominant, moderate-to-severe bleeding syndromes [9].

The second one is Fetal/Neonatal Alloimmune Thrombocytopenia (FNAIT). Some natural missense mutations at specific positions in α_IIb_β_3_ neither affect the expression nor the function of the protein, but define Human Platelet Alloantigens (HPA) systems. Fetal/neonatal platelets are destroyed by maternal antibodies in mothers who lack an HPA allele inherited from the father. FNAIT clinical consequences range from no symptoms to intracranial hemorrhages with a risk of neurological sequelae and/or fetal/neonatal death [10,11]. Up to now, 35 HPA systems and alloantigens are described (formerly at EBI, now at https://www.versiti.org/medical-professionals/precision-medicine-expertise/platelet-antigen-database#hpa-database, accessed on 16 November 2021). The effects of these amino acid substitutions on α_IIb_β_3_ structure remain largely unknown.

Our research group has been interested in understanding how amino acid substitutions in GT can structurally impact α_IIb_β_3_. As integrins have a large size and complex organization, a limited number of structures have been resolved so far. Strikingly, they all have impressive conservation of protein folds, even if they all are evolutionary distant and associated to different biological pathways. Using whole α_IIb_β_3_ ectodomain (PDB id 3FCS [4]) in closed conformation, we have showed that the amino acid variant located in β_3_, Lys253Met (K253M), generated by a GT mutation, impaired key ionic interactions between the α_IIb_ β-propeller and the β_3_ βI-like domain [12].

HPA-1 is the most frequent HPA associated to FNAIT in the Caucasian population. It is associated with an amino acid polymorphism located in β_3_, Leu33 and Pro33; it defines the HPA-1a and 1b alleles, respectively. Interestingly, a very rare third isoform also exists, i.e., Val33. Molecular dynamics (MD) simulations were used to compare the structures of these isoforms. This position remained surprisingly rigid inside a larger deformable region, underlying the possibility to express a specific epitope. The P33-β_3_ variant presented a higher mobility and specific conformations of *I-EGF-1*, *I-EGF-2* and *PSI* domains [13], showing that V33 isoform behaves as an intermediate between L33 and P33 [14].

Pagani and collaborators show that, under flow-dynamic conditions, the P33 variant displays prothrombotic properties, with increased platelet adhesion and aggregation, thrombus formation, and outside in signaling the triggering of the opening of α_IIb_β_3_ structure. MD simulations of the ectodomain of the L33 and P33 isoforms underlined that the P33 weakens interdomain interactions at the *knee* in regards to L33 dynamics, and alters the structural dynamics of the integrin to a more unbent and splayed state [15].

Mansour and collaborators were interested in the mechanism of deformation of its structure in response to the presence of the Glanzmann N2D mutation located in the β-propeller domain of the α_IIb_ chain. This mutation induces the loss of α_IIb_β_3_ expression at the platelet surface. The latter affects a Ca^2+^ binding site that plays an important role in the conformational stability of the integrin and its affinity for the ligand [16]. Laguerre and collaborators focused on the β_3_ P163S GT variant and underlined a change in the potential interface with α_IIb_ [17]. Nurden and collaborators studied missense mutations of β_3_ involved in GT and showed that it affects the structure of the two chains: α_IIb_ and β_3_. These changes correspond to the loss of molecular interactions that lead to structural changes. However, these in silico analyses had their limitations due to (i) very short simulation times, (ii) often conducted using a single simulation, and (iii) the absence of ions essential for local protein structures structuring [18].

**Figure 1:**
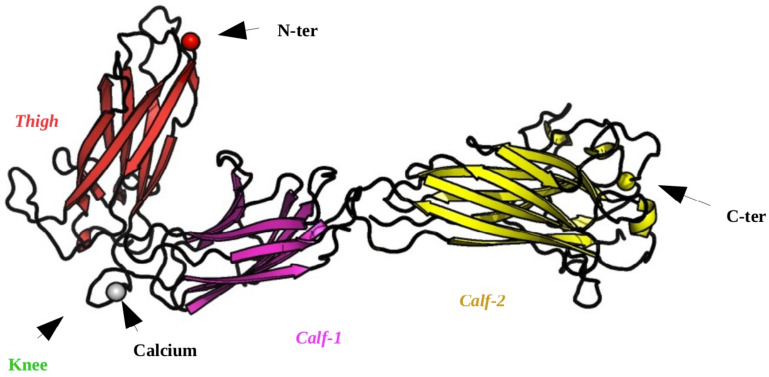
The three domains of integrin α_IIb_β_3_: *Thigh*, *Calf-1* and *Calf-2*. It is visualized with PyMOL [20]. The *Thigh* domain is colored in magenta, *Calf-1* in purple and *Calf-2* in yellow. The *Knee* region is colored dark green with Ca^2 +^ ion represented by a grey sphere. The N-terminus is indicated by a red sphere, and the C-terminus by a yellow sphere.

The lower leg domains in the α_IIb_ subunit, *Thigh*, *Calf-1*, and *Calf-2* (see Figure 1), have a conserved immunoglobulin fold, which is well known for its rigid β-sheet structure. Thus, it makes them ideal for analyzing the impact of GT variants on the local structure. Using new strategies in MD, in 2017, we studied the effect of seven variants of the α_IIb_ *Calf-1* domain, which are known to impair α_IIb_β_3_ integrin expression in GT. Surprisingly, at the aa (amino acid) variation/substitution positions, no difference in terms of local conformations was observed for these variants. On the other hand, some loops (mainly loops 2 and 8) were strongly impacted in their dynamics for all these variants, leading to the question of allostery/long-range interaction [5].

A similar analysis with a new GT variant G540D of β3 *I-EGF-3* discovered in Turkish families revealed a strong impact in terms of dynamics. The GT variant showed disorder tendencies in the second part of *I-EGF-3* whereas it was rigid or slightly flexible at most [19].

**Figure 2:**
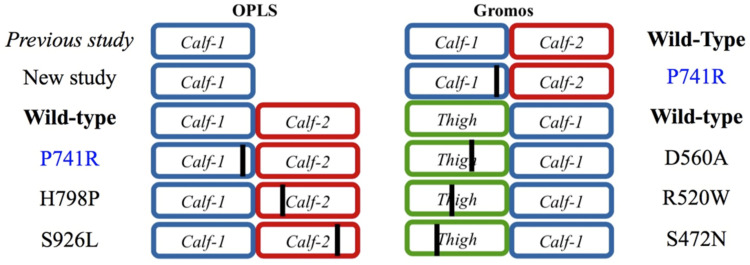
The 11 new systems studied. The black line indicates the position of the mutation.

As mentioned above, MD simulations of isolated α_IIb_ *Calf-1* domain showed that seven GT variants have distant structural impacts instead, i.e., long-range allosteric effects. In the present study, we extend the analysis of the *Calf-1* domain with its adjacent domains. Firstly, the potential impact of the forcefield (*ff*) is evaluated. Then, the neighboring domains from the large structural assembly of integrin α_IIb_β_3_ are added to *Calf-1*. Studying the *Calf-1* + *Calf-2* and *Thigh* + *Calf-1* domains together also tests whether the clipping had an effect on the analysis previously conducted with the isolated *Calf-1* (see Figure 1). Finally, different GT and HPA variants are modelled into these rigid domains and each mutation is studied for its potential effect on the local and distant structures. All the MDs performed are of equivalent length and can therefore be compared (see Figure 2 for a description of the 11 new systems).

## 2. Results

### 2.1. Structural Modeling of the Calf-2 Domain

In this study, the *Calf-2* domain (residues 744 to 952) has to be completed as the structural information for 2 regions is missing in the selected template (unresolved in the crystallographic structure). The first region is 11 residues long (positions 763–775) whereas the second one is 34 residues long (positions 840–873). The second region is long enough to be beyond the scope of default loop-modelling algorithms [21], with no repetitive secondary structure [22,23] and high flexibility content [24,25,26]. To expertly model these, sequence homologs were mined using PSI-BLAST [27] and structurally similar molecules were identified using the FATCAT algorithm (Flexible structure AlignmenT by Chaining Aligned fragment pairs allowing Twists) [28]. Models were generated with MODELLER. After carefully selecting the templates, additional distance restraints were added to ensure that loops were not directly interacting with the rest of the structure. All the generated models were inspected manually and, finally, the model that was least impacted by completion had a low Root Mean Square Deviation (RMSD) to the template and the correct loop geometry was selected. This model has an excellent overall RMSD value of 0.2 Å with X-ray structure (see Figure S8 for more details and Figure S1).

### 2.2. Designing the Calf-1 + Calf-2 Simulation System

#### 2.2.1. Molecular Dynamics

As described above, in a previous study, *Calf-1* was also analyzed for GT variants, but without any other partner. Moreover, the previous study was conducted with an older version of Gromacs. Therefore, at first, the 850 nanosecond simulations were performed again to ensure the quality of our planned experiments (see Figure 2). No difference was spotted in the Root Mean Square Fluctuation (RMSF) of the *Calf-1* domain simulated under the newer version of Gromacs and the older version. Moreover, the upgraded simulation setup of 1 microsecond also did not show any significant impact (see Supplementary Materials for more details).

**Figure 3:**
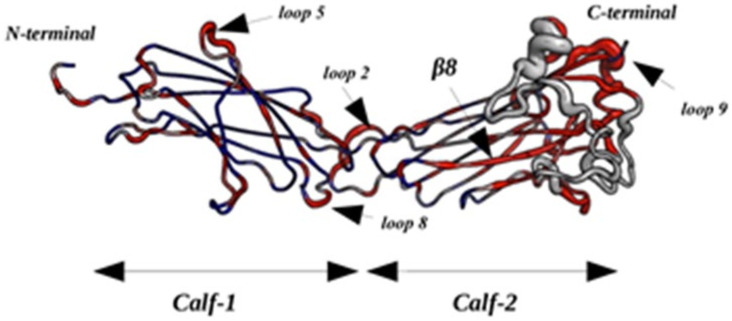
Representation of the *Calf-1* and *Calf-2* domains according to the mobility of atoms, represented by B-factor values. They range from less mobile (thin line, blue) to more mobile (thick coating, red).

After acquiring confidence in the structural model of *Calf-1* + *Calf-2* domains, their validation was also performed using MD simulations followed by associated analysis. In order to compare the RMSF of the simulated *Calf-1* + *Calf-2* system, at first the B-factor profile for the same system was studied (as shown in Figure 3). It is worth mentioning that B-factors are the thermal factors derived from the X-ray data and are directly proportional to the mean square displacement of an atom. Since the RMSF also takes into account the root of mean square fluctuation of an atom, it can be compared to the B-factor values to assess the flexibility profiles.

For *Calf-1* and *Calf-2* domains, these values show that regions associated with high values are located at the loops connecting the β-strands. As per expectations, the β-sheets are on average rigid, thus forming the core of the immunoglobulin fold in *Calf-1* and *Calf-2*. The *Calf-1* domain has 4 regions with high flexibility at loop 2 (620–621), loop 5 (667–670) and loop 8 (711–712). These values of the *Calf-1* domain are in good agreement with the RMSF values calculated in the isolated analysis of the *Calf-1* domain. In contrast, *Calf-2* is much more flexible at these β-strands: β-strand 8 (positions 898–908) shows high values of B-factors, thus indicating some disorder in the local structure. In addition, the loops closer to the ones located just before the membrane show very high values of B-factors, especially at loop 9 (901–921). In addition, the *Calf-2* loops that have large missing regions have very high B-factor values for the modeled residues, making their study under a simulated environment much more stimulating.

#### 2.2.2. Assessing the Impact of the Adjunction of the *Calf-2* Domain to *Calf-1*

A logical question that arises with such kind of analysis is the potential effect of cutting structural domains and studying them in isolation. In order to address it, the dynamic behavior of the *Calf-1* domain was compared with the neighboring *Calf-2* domain to see if the inclusion of the latter leads to any changes in the dynamics of *Calf-1*. In our analysis, *Calf-1* is the only domain that was studied in isolation as well as in combination with *Calf-2*.

**Figure 4:**
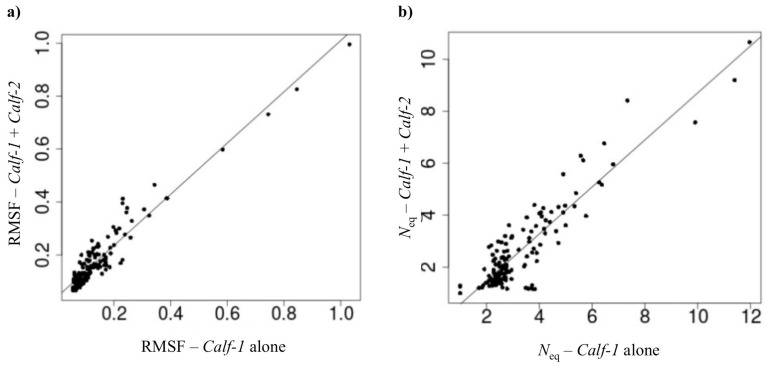
*Calf-1* vs. *Calf-1 + Calf-2*. (**a**) RMSF values of *Calf-1* alone and of the *Calf-1* & *Calf-2* system. (**b**) Same representation for *N*_eq_ values.

Figure 4 summarizes this by comparing RMSF and a protein block entropy value (see Section 4.4), namely *N*_eq_, values of both systems. RMSF is often used to determine flexible mobile regions; generally, they correspond to loops. Compared to the behavior of *Calf-1* alone, the most mobile regions are similar; however, the mobility is rather amplified for the *Calf-1* & *Calf-2* system. This can be explained by the inclusion of highly mobile region of loop 9, which can impact the mean values. Nonetheless, this difference does not change the general behavior of the domain. Indeed, the correlation in RMSF values is extremely high (0.96, see Figure 4a) and such is also the case with the *N*_eq_ values (0.91, see Figure 4b). It is noteworthy that the ΔPB values (this measure corresponds to the sum of difference between protein block distribution observed at one position, see Section 4.4, Equation (2)) show variations in areas 622–625 (loop 2), 639–641 (loop 3), 712–715 (loop 8) and 726–728 (loop 9). Compared to the B-factor values, these variations are consistent with loops 2 and 8. The positions can slightly vary; hence, loop 3 is not associated with a high B-factor value. However, these mobile regions are observed whether *Calf-1* is associated with *Calf-2* or not. Thus, it can be concluded that the dynamics of the two systems are comparable, and the association of *Calf-1* with *Calf-2* does not significantly impact the behavior of *Calf-1* dynamics.

#### 2.2.3. MD Simulations: Potential Impact of the Change in Forcefields

**Figure 5:**
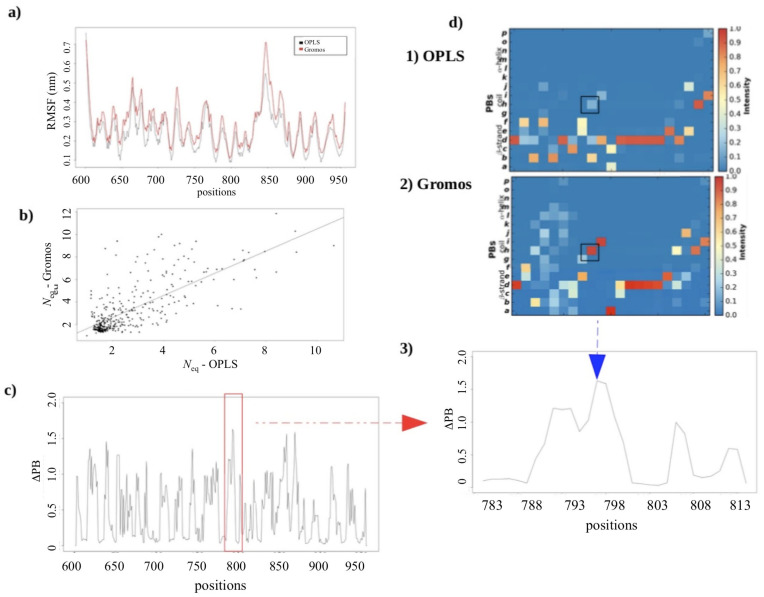
OPLS ff vs. Gromos ff. (**a**) RMSF and (**b**) Correlation between the *N*_eq_ values of each residue in *Calf-1* simulated with OPLS *ff* (*x*-axis) to *Calf-1* simulated in Gromos *ff* (*y*-axis). (**c**) ΔPB between the simulations performed in these two forcefields. (**d**) PBs occurrence map [29] of positions 783 to 813 for (1) OPLS *ff*, and (2) Gromos *ff*, with corresponding (3) focused ΔPB profile.

Another classical question is the possibility to have an impact of the selected forcefield on the simulation experiments. Although it is an open-ended debate, the effects have to be tested as we have been dealing with multiple simulation experiments performed on different simulation systems (the *Calf-1*, *Calf-1* + *Calf-2*, *Thigh* + *Calf-1;* see Figure 2) over a spectrum of time. Therefore, to address this issue, the *Calf-1* simulation experiment performed in Gromos *ff* was compared to the results obtained from *Calf-1* simulation experiment performed in OPLS-AA *ff*. Figure 5a shows that the RMSF variations are almost identical; the difference in RMSF is on average only 0.24 nm with a correlation of 0.96. Figure 5b shows the correlation of the values of *N*_eq_. The correlation is comparatively weaker (0.73), but more precisely, the same moving zones are identified for the two forcefields. Therefore, the use of protein blocks allows a more precise analysis than the classical RMSF.

ΔPB shows that there are more than 20% of the residues with a ΔPB greater than 1 (see Figure 5c). These regions correspond to positions 620–630 (loop 2), 640–645 (loop 3) and 652–657 (loop 4) of the *Calf-1* domain and positions 742–747 (loop 1), 761–775 (loop 3), 788–795 (loop 4), 850–862 (start of completed loop 7), 865–875 (end of completed loop 7), 910–925 (loop 9) and 931–945 (loop 10) from *Calf-2*. These variations are localized within the loops of both domains. All these positions showing significant variations correspond to transitions between PBs *b* (N-cap of β-strands) and *c* (β-strands) and PBs *h* and *i* (i.e., PB loops). To understand the observed higher ΔPB values, focused profiles were analyzed for the region 783–813, which forms the interface of *Calf-1* and *Calf-2* and lies just upstream of the modeled loop in *Calf-2*.

Figure 5d shows an example of these changes of PBs (heatmaps) and the difference between the two forcefields in terms of frequency. Each pixel in the heatmap depicts the frequency of the conformation acquired at that position over simulation time: red indicates higher frequency whereas blue shows lower frequency in a spectrum from red to yellow to blue (see also Figure S2). The interesting case of residue 796 (framed in black), representing the greatest value of ΔPB (≈1.6), shows a difference between the types of PBs. Indeed, for residue 796, with the use of the OPLS *ff*, the occurrence of PBs *b* (N-cap of β-strands), *d* (core of β-strand) and *h* (loop) is 60%, 20% and 20%, respectively, which would indicate a rather extended structuring, whereas with the use of Gromos *ff*, the proportion of PB *h* is 100%, i.e., a true loop region. So, for this residue, the conformation is different according to the forcefield, i.e., difference of 80% for the PB *h*. A plausible explanation can be the positioning of the residue right at the beginning of the β-strand. Nonetheless, no real difference in β-sheets (rigid) is observed, except for a slight fluctuation between the two main PBs identifying the core β-strand (PBs *c* and *d*). In conclusion, the main behaviors are not impacted by the change in forcefields of simulation systems under study in this paper.

#### 2.2.4. Dynamics of Expertly Modeled Loops of the *Calf*-2 Domain

**Figure 6:**
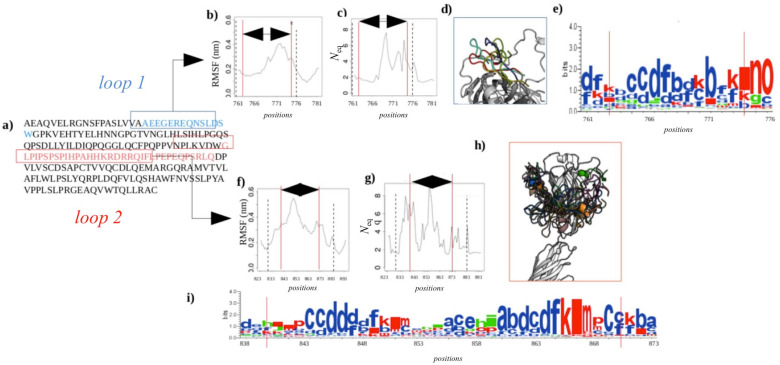
*Calf-2* completed loops. (**a**) Sequence of the *Calf-2* domain: in blue: the first completed loop; in red: the second one. (**b**,**f**) show the RMSF values for two loops. (**c**,**g**) show the *N*_eq_ values for the two loops. (**d**,**h**) are the 3-D representation of different conformations of the two modeled loops. (**e**,**i**) show the conservation logo maps of these two loops. The red lines on the graphs indicate the boundaries of the completed loops and in black dot lines the limits of entire loops.

After addressing the concerns about the selective bias in our designed simulation experiments, it is particularly interesting to analyze the behavior of modeled loops (positions: 763–775 for *Calf-2′s* completed loop 1 and positions 840–873 for loop 2 numbered as they were modeled; see Figure 6a). In the overall simulation system, these correspond to loop 3 and loop 7, respectively.

The values of RMSF for loop 1 are quite high and even higher for loop 2 (see Figure 6b,f). Figure 6e,i represent the distribution of PBs observed at each position. The height of letters indicates the relative frequency of PB at that position, i.e., one big letter means only one type of PB, and small and numerous letters show higher diversity. *N*_eq_ values have been scaled in regard to flexible and disordered regions [30,31]. *N*_eq_ value higher than 8 is considered as disordered position, whereas values less than 8 but greater than 6 are considered highly flexible, and values around 4 show inherent flexibility. Figure 6c,g show that these loops are not completely disordered, but they have specific regions. In regard to a specific rigid region encompassed between highly disordered regions [5,31], they were analyzed in depth [32]. The first completed loop begins with a slightly deformable region (before position 767, *N*_eq_ is around 2), before going to the restricted disorder position 768, and the rest of the loop is between flexible and highly flexible. The second loop, the longest one, has more highly flexible parts, and few disordered ones, i.e., positions 837 (in the resolved one) and position 865. Interestingly, this long loop encompasses two small, rigid and slightly deformable regions (around positions 845 and 867). The 3D visualization shows some examples of these important sampling (see Figure 6d,h). RMSF and *N*_eq_ contrasts highlight the existence of locally rigid zones in these loops. Hence, Figure 6e,i show that regions 771–772 and 773–774 of loop 1, and 844–846 and 865–867 of loop 2 mainly have PB *d* signature corresponding to extended regular structures. These regions are influenced by the dynamics and the great flexibility of their surrounding loops, i.e., PBs b, *f*, and *k* are observed for the two loops indicating these areas are much more deformable (as seen in [33]).

### 2.3. Modeling Punctual Mutations to Generate Structural Variants of Interest

#### 2.3.1. Analyses of the Variants

Out of the multiple *Calf-2* and *Thigh* domain variants that are related to GT, a selected few were tested. These were selected based on their potential impact on the subunit structure. The two *Calf-2* variants generated by two independent punctual mutations S926L and H798P [12] prevent the expression of integrin complex at the surface of COS-7 cells by altering the α_IIb_ subunit structure. The variant D560A is referenced in the dbSNP (Single Nucleotide Polymorphism database, Acc: rs778608263) with a PolyPhen score of 0.999 indicating that the substitution is highly deleterious. The R520W variant of the *Thigh* domain and was detected in Indian patients who had GT [34]. Its deleterious effects have been confirmed by in vitro studies. The PolyPhen score for this variant is 1, indicating a highly negative impact on its phenotype. Finally, the S472N variant located in the Thigh domain was selected as a control to our simulation experiments. This mutation does not contribute to GT, but rather induces fetomaternal alloimmunization (HPA-24). It has a PolyPhen score of 0 and the expression of α_IIb_β_3_ is unaffected, thus structural impacts are expected to be significantly minimal.

#### 2.3.2. Assessing the Structural Impact of the GT Variant P741R

The variant P741R is located at the end of *Calf-1* and almost at its junction with the *Calf-2* domain. At this position, a Proline residue that is known for introducing kinks in the backbone is substituted by a positively charged Arginine. The previous study carried out with an isolated *Calf-1* domain did not allow to conclude whether the allosteric effects observed were a result of the P741R substitution or a bias introduced by the absence of the *Calf-2* domain. As explained in Sections above, a very good correlation is found between the RMSF values for the *Calf-1* system and *Calf-1* + *Calf-2* system (0.95, see Figure S3a). Interestingly, local variations are found at the same place for both systems. Moreover, the values of *N*_eq_ show an excellent agreement between the 2 systems with a correlation of 0.90 (see Figure S3b). PB *d* (core of β-strand) is found in same proportion, 0.9, which indicates that the association of *Calf-2* to *Calf-1* has no quantifiable impact on the results obtained previously with an isolated *Calf-1* domain (see Figure S3c–f).

**Figure 7:**
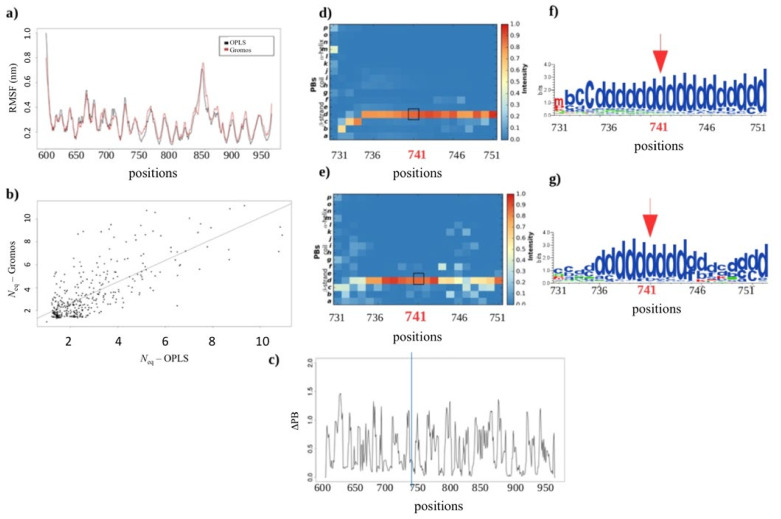
P741R variant. (**a**) RMSF and (**b**) *N*_eq_ for the simulations with two forcefields (OPLS in red, Gromos in black). (**c**) Corresponding ΔPB between the two forcefields for the variant, with zoom on position 741. (**d**,**e**) depict the PB maps for residue 741 of the wild-type system and for the variant respectively (see also Figure S4), and (**f**,**g**) show the corresponding conservation Logo. The squares in black, the arrows in red and the line in blue mark the position of residue 741.

It is therefore possible to analyze the structural effects of the P741R variant using either of the two forcefields. Figure 7a shows how close the RMSF values obtained are using the two forcefields for Arg741 MD simulations; the correlation equals to 0.94 underlying the excellent similarity between the results obtained with the two forcefields. *N*_eq_ correlation is observed to be slightly lower, i.e., 0.74 (see Figure 7b), but is expected. The PBs’ heatmap shows a slight dispersion of PB *d* towards PBs *b*, *c*, *l*, and *m*, especially at the extremities of repetitive structures that represents connecting loops. Hence, 20% of the residues show significant local variations in the loops, with a value of ΔPB greater than 1 (see Figure 7c). This indicates a difference in number of occurrences of PBs. Regarding the P741R substitution, the main PB is PB *d* observed in both the cases. With the use of OPLS *ff*, its occurrence is approximately 100%, whereas with Gromos *ff*, it is 90% (see Figure 7d–g), which is not a significant difference. This shows that selected forcefield does not affect the local structure dynamics for the P741R variant.

#### 2.3.3. Analysis of the GT H798P and S926L Variants of the *Calf-2* Domain

To go further downstream towards *Calf-2* domain, two new systems containing substitutions associated with Glanzmann thrombasthenia were simulated, i.e., H798P (His to Pro) and S926L (Ser to Leu). The variant H798P arises due to a substitution of the Histidine residue at position 798 by a Proline. The side chain of Proline interacts with its own backbone, thus limiting the possibility of other side-chain-to-side-chain interactions. Thus, the substitution can have a substantial effect on backbone conformation. However, the substitution of a polar Serine residue by non-polar Leucine at 926 may not have a significant structural impact on the backbone.

The comparison of RMSF observations (see Figure S5) show the exceptionally similar flexibility profile of the variants and the wild-type system. The most important variations are in the loops of the two domains: residues 620–623 (loop 2), 667–669 (loop 5), 711–713 (loop 8) and 731–733 (loop 9) of the *Calf-1* domain, in the two modeled loops (completed loop 1: 763–775 and completed loop 2: 840–873) and regions 784–786 (loop 4), 919–921 (loop 9) and 934–35 (loop 10) at the *Calf-2* domain. The variants do not undergo much change in their conformations.

##### Local Structural Changes Due to H798P

**Figure 8:**
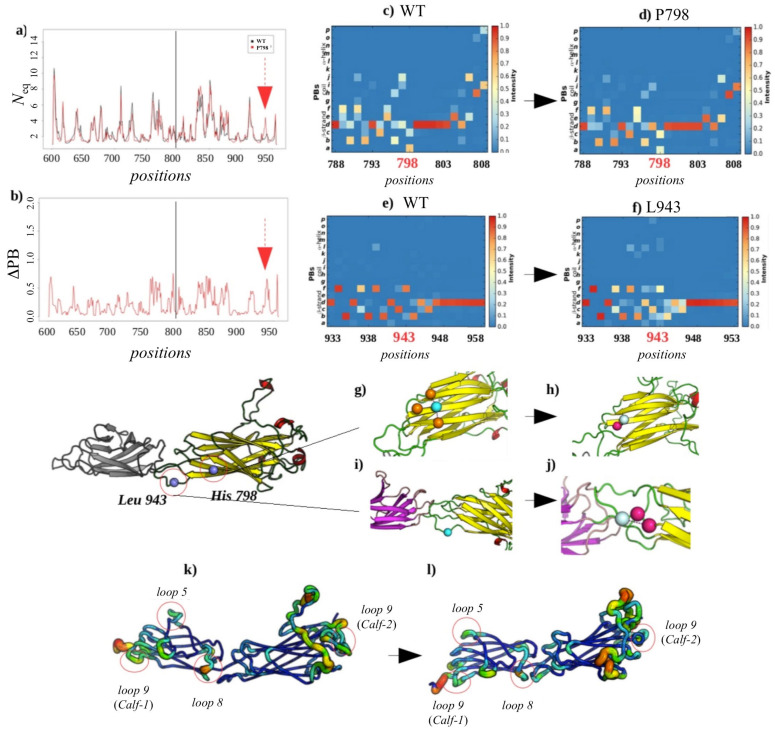
H798P GT variant. (**a**) *N*_eq_ of the wild system (*H798*, color in black) and GT variant (*P798*, color in red). (**b**) ΔPB between the two systems. The mutated residue is indicated by a black line, and the residue with the strongest ΔPB by arrows. PB occurrence maps are shown around position 748 and 943 (**c**,**f**) for the wild-type and (**d**,**e**) for the variant. (**g**) Interactions of residue H798 (cyan) and (**h**) of residue P798 (light cyan); in orange, the residues interact in the same way in the wild-type and the variant; in violet, the new interacting residue. (**i**) Same representation for the residue L943 in the wild-type and (**j**) for the variant. Representation of the *Calf-1* and *Calf-2* domains according to the flexibility measured by values of *N*_eq_ for (**k**) wild-type and (**l**) for the variant, moving from the least flexible (thin line, blue) to the most mobile (thick line, red). The most mobile parts representing high values of *N*_eq_ are circled in red.

Figure 8 summarizes the main information on the comparison for this system. The distribution of RMSF and *N*_eq_ values show a good correlation (0.97 and 0.98, respectively). The most important variations are observed in the loops and especially in the two that were modeled. Compared to the WT system, the differences in *N*_eq_ are found at residues 669–670 (loop 5), 713–715 (loop 8) and 728–730 (loop 9) of the *Calf-1* domain.

A slight decrease in the flexibility of these loops is observed due to the variant’s impact. However, a different trend is observed in *Calf-2* at the level of two modeled loops (loop 1: 763–775 and loop 2: 840–873) and 916–918 (loop 9). These regions are inherently flexible, and their flexibility increases due to the impact of these variants (see Figure 8k,l). In contrast to our expectations, a large proportion of the residues, including the variant impact site H798P, show low values of RMSF and *N*_eq_. Indeed, the Δ*N*_eq_ of 0.2 also indicates that H798P position does not undergo any change compared to the wild system, and also represents a very low ΔPB of 0.02 (see Figure 8b). The observation of PB occurrence maps from the wild system and that of the variant shows that the PBs predominantly present are PBs *a* (N-cap of the β-strand) and *c* (core of β-strand). It is noteworthy that there is a conservation of local structure at the position of this punctual mutation and the statistics indicate towards enhancement of β-strand formation (see Figure 8c,d). Such behavior is not unexpected as the β-strand formation is maintained by backbone hydrogen bonding and the substitution does not alter those interactions. On the other hand, the hydrogen interactions and ionic effects produced by Histidine disappear with the substitution by a Proline. Moreover, Proline, a non-polar amino acid, establishes a single hydrophobic bond with Leucine 797. Nonetheless, these interaction changes do not alter the structure of the domain at the variant site and indicates towards a conformational compensation mechanism.

##### Non-Local Impact of the Single Amino Acid Variation in the *Calf-2* Domain

As it can be seen in Figure 8c,d, the PB profile does not change a lot between the wild-type and the variant. Rather, at the substitution site, H798P, the color changes indicate more rigidity instead. Therefore, it was interesting to observe structural changes that appeared at a distant site to the mutation site. Figure 8e,f highlights a residue Leucine 943 located in loop 10 of the *Calf-2* domain that presents highest values of *N*_eq_ and ΔPB of the system, 2.3 and 2.0, respectively. The majority PB *f* (C-ter of the β-strand) becomes less frequent in the variant and frequency of PBs defining helical turns begin to appear (PB *l* and *m* observed). Similar trends can be seen in the near vicinity of this site (Leu 943, color intensity of decreases from dark orange to pale yellow). A close inspection into the structure reveals that, whereas this residue shows no interaction in the wild-type, it establishes two new hydrogen-bond interactions in the variant. These interactions result in loss of PB *f* (50%) and a set of PB *a* and *b* (N-cap of β-strand) in equal proportion. The gained interaction of Leucine changes the PBs profile of this region by reducing the proportion of PB *f*.

Two other interesting cases located on the *Calf-1* domain were studied: Glycine 714 and Asparagine 732. They are among the residues that show high values of *N*_eq_. The residue Gly714 exhibits, in the manner of the residue Leu943, stability at the level of its local conformation despite having differences in interactions in wild-type and variant systems. Indeed, it establishes a hydrophobic bond that reinforces the appearance of the PB *g* (loop) in the variant. On the other hand, the residue Asn732 does not show any change of interactions nor of structural conformation. Indeed, the ΔPB is very low (0.1). This residue shows the same local conformations in the wild and the variant system whereas maintaining high mobility, which remains similar in both.

##### Local and Non-Local Structural Impacts Due to S926L

**Figure 9:**
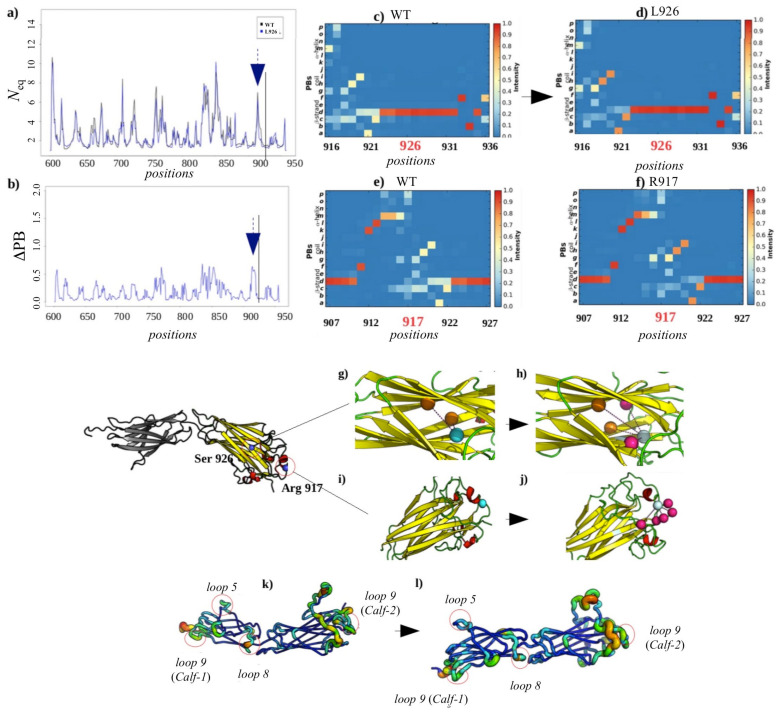
S926L GT variant. (**a**) *N*_eq_ of the wild system (*S926*, color in black) and GT variant (*L926*, color in blue). (**b**) ΔPB between the two systems. The mutated residue is indicated by a black line, and the residue with the strongest ΔPB by arrows. PB occurrence maps are shown around position 926 and 917 (**c**) and (**f**) for the wild-type and (**d**) and (**e**) for the variant. (**g**) Interactions of residue S926 (cyan) and (**h**) of residue L926 (light cyan); in orange, the residues interact in the same way in the wild-type and the variant; in violet, the new interacting residue. (**i**) Same representation for the residue R917 in the wild-type and (**j**) for the variant. Representation of the *Calf-1* and *Calf-2* domain according to the flexibility measured by values of *N*_eq_ for (**k**) wild-type and (**l**) for the variant, moving from the least flexible (thin line, blue) to the most mobile (thick line, red). The most mobile parts representing high values of *N*_eq_ are circled in red.

The second GT variant is generated by a substitution of Serine residue at position 926 by a Leucine. The patterns in *N*_eq_ variations (comparison to WT) are similar to those observed in the H798P variant. Indeed, these variations affect residues 669–671 (loop 5), 711–713 (loop 8) and 729–731 (loop 9) of the *Calf-1* domain, the two modeled loops (1: positions 763–775 and 2: 840–873) and residues 916–918 (loop 9) of domain *Calf-2*. These regions are observed to be even more mobile in this variant. The mutated residue does not undergo change in terms of *N*_eq_ (see Figure 9a) and retains local conformational stability with a low ΔPB of 0.07 (see Figure 9b) having a high occurrence of PB *d* (β-strand element, see Figure 9c,d). Hence, no difference in local conformations for this residue is observed between the wild-type and mutated forms. On the other hand, a hydrophobic interaction appears in the variant (see Figure 9h), thus reinforcing the structuring of this region into a β strand. The Arginine 917 located ten residues upstream of the mutated residue exhibits a higher ΔPB of 1.5 (max. value 2.0). This is a strong destabilization, which is observed particularly by the low frequency of PBs *p*, *o*, *m*, *d* and *c*. Hydrogen interactions that were not present in the wild-type appear in the variant. These alter the local conformation at the residue 917. Appearance of a PB *h* to the disadvantage of PB *d* can be seen in Figure 9e,f, thus pushing the local structure into coiled conformation. The Glycine 714 and Asparagine 732 residues studied in the H798P variant of the *Calf-1* domain were also analyzed in the case of the S926L variant as they again present high *N*_eq_ values in this case. As in the H798P variant, S926 also shows low ΔPB indicating that the local structure at the mutated site is conserved. However, increase in flexibility is observed at distant sites indicating a compensatory effect to adjust the impact on structural mutation sites.

Thus, the analysis of the different GT variants in context of *Calf-2* shows a more complex story than for those analyzed in *Calf-1* previously. Indeed, the mutated residues are, as for *Calf-1*, still particularly stable and above all comparable to their WT equivalent. *Calf-1* showed long-range implications in a quasi-systematic way. However, in *Calf-2* domain, such effects are tenuous but do exist. The variants affected the modeled regions and a small loop with varying intensities; however, in the isolated *Calf-1* study, these effects were quite prominent. This analysis also shows the impact of the variants on the *Calf-1* domain. Although the impact is very small, the long-range effects of mutations in the structure of integrin α_IIb_ are again underlined.

#### 2.3.4. Dynamics of *Thigh* and *Calf-1* Domains

In this last part, the regions upstream of the *Calf-1* domain were analyzed. Between the *Thigh* and *Calf-1* domains is located a short region called “knee”. Being highly flexible, this short region acts as a joint and is considered crucial for the opening of the structure of the α_IIb_ chain [35] during the hemostasis process. In this region, the crystallographic structures reveal the presence of a Ca^2+^ ion (see Figure S6c). Therefore, Gromos *ff* was used, as Ca^2+^ was better parameterized in this forcefield. Four systems were used for MD simulations (the wild-type and three structural variant systems: D560A, R520W and S472N). The analysis of the MD simulations of these systems shows a high stability for both for the temperature and the pressure expressed as energies (potential, kinetic and total). The only surprise comes from the displacement of the Ca^2+^ ion out of its initial place during some dynamics.

As per the crystal coordinates, Ca^2+^ ion binds to the four residues Cys 602, Asp 605, Val 607 and Glu 642 (see Figure S6c). Interactions with these four residues were identified as “borderline” (weak bonds) or even “outlier” by the web server CheckMyMetal [36]. The web server highlights the possibility of binding with water molecules. The residue coordinates involved in the interaction with Calcium ion are not all conserved within 11 simulations (see Figure S6a). The interactions with residue Val 607 are maintained in all systems with a high proportion of 84.5% of simulation time. The Asp 605 residue is found to be involved with Ca^2+^ in ~65% of the simulation time. On the other hand, the proportion becomes lower for the Glu 642 residue and even lesser for Cys 602 (32% and 9% respectively; see Figure S6b). These results were found to be consistent with a similar study [37,38].

The most mobile regions, for the -d-type and the three variants, were identified using RMSF (see Figure S7); they corresponded to loops. A correlation of 0.98 exists between the RMSF values of the wild-type and each of the three variants. The most mobile regions, for the *Thigh* domain, are located at the residues 480–482 (loop 1), 499–501 (loop 2), 540–542 (loop 6) and 580–582 (loop 8). For metal–residue interactions located in the *Calf-1*, the most mobile regions were identified in 626–628 (loop 2), 681–683 (loop 6) and 716–718 (end of loop 8). The average Δ*N*_eq_ shows that the local structural mobility is slightly enhanced at the *Calf-1* domain than at *Thigh* domain for the D560A variant (0.45 and 0.56, respectively). Similar values were observed for the simulations of R520W variant (*Calf-1*: 0.42 and *Thigh*: 0.48). The S472N variant system does not have differences in the two domains; the two Δ*N*_eq_ averages are quite close with 0.4 for the *Calf-1* domain and 0.35 for the *Thigh* domain.

Concerning D560A, the mutated residue is located in a flexible region. ΔPB equals to 0.8. The main PB is PB *p* (end of α-helix) for the wild-type and the variant, (60% and 70% respectively), i.e., only the less frequent conformations change in the variant system. The substitution of Aspartate by an Alanine results in the loss of an ionic bond. However, this does not change the PB profiles from the wild-type system given that the Asp560 is located in an already flexible region. Amongst other changes induced by this substitution, Glutamate 711 located in *Calf-1* loop 8 shows a Δ*N*_eq_ of 3.59, the highest for this system, and is associated with the maximum ΔPB of 0.91. This region is one of the most mobile ones counted among the top 5% of the most fluctuating regions in all the systems under study. The main PB in the wild-type is PB *f* (end of β-strand) with a proportion of 50%, in the variant; this frequency drops to 20% with an increase in the PB *k* (start of the helix) to 30%. This can be visualized in the variant by the appearance of new hydrogen interactions in the variant when compared to WT.

For the second one, R520W, the mutated residue does not undergo any change in *N*_eq_ compared to the wild-type system; its Δ*N*_eq_ is 0.18 and has a ΔPB of 0.50. The variant has slightly more helical conformation with a proportion of PB *m* (helical core) that increases from 30% in the wild-type system to 50% in the variant. In counterpart, the proportion of PB *f* (after of β strand) decreases from 50% to 40%. The substitution of an Arginine by a Tryptophan makes it possible to create hydrophobic and aromatic interactions to compensate the loss of both ionic bonds at positions D560 and R520. In the *Thigh* domain, Glutamine 514 in loop 3 shows the highest Δ*N*_eq_ of 3.81 of this system and is associated with the maximum ΔPB of 1.18. This region is mainly formed by an α-helix in the variant with a large proportion of PB *k* (90%) compared to the wild-type (40%). The gain of a second hydrogen bond allows the strengthening of the α-helical structure in this region.

The third variant system contains a polar Serine substituted by polar Asparagine at position 472. The variant S472N is not associated to GT, but to the blood group HPA. Local conformations at this position are quite similar with majority PBs *b* and *i*, resulting in a low ΔPB of 0.20. The substitution does not induce any conformational change. However, gain of two more hydrogen bonds by Asparagine substitution makes it possible to maintain a similar PB profile. Among the distant positions, Glutamate 578 of loop 7 (*Thigh*) shows a maximum ΔPB of 1.28, which should indicate a spike in flexibility. However, this residue is found in a naturally flexible region. The majority block is *i* and is present more in the wild-type form (50% vs. 30%). There is a loss of helical structure with an increase in the frequencies of PBs *f* and *e* (after β-strand) in the variant. The correlation between the *N*_eq_ values of the wild-type form and those of the variants D560A, R520W and S472N is 0.93, 0.90 and 0.95 respectively, i.e., there is no significant difference between wild-type and variants.

The three variants do not undergo change in their local conformations compared to the wild-type. This study shows that the punctual mutations investigated have little or no effect on the structural conformations locally and/or in the immediate environment of the impacted sites. On the other hand, the MDs reveal that some regions distant from mutation sites may be affected (large increases in *N*_eq_). These allosteric phenomena concern all the domains studied to date, the *Calf-1*, *Calf-2* and *Thigh* domains, either alone or together. The deformability observed is mainly localized in the loops connecting the β-strands forming the core of the immunoglobulin fold. Finally, our study suggests that these allosteric mechanisms occur regardless of the clinical context, GT or HPA polymorphisms.

## 3. Discussion

The objective of this study was to look at the effect of amino acid substitutions (missense mutations) on the structure of integrin α_IIb_β_3_ in pathological contexts linked to Glanzmann thrombasthenia or in fetomaternal alloimmunization following our previous research on *Calf-1* alone. We have previously shown that the mutations (i) did not change the local protein conformations, and (ii) did not change their dynamics whereas (iii) the effects were always found far away from these sites displaced to the connecting loops [5].

To proceed in a systematic manner, we decided to analyze the potential effect of forcefields on the simulated systems. Observation of the dynamics of the *Calf-1* domain through the prism of the protein blocks showed subtle changes between the two simulations performed using two different forcefields. These differences are small, but not negligible, as they mostly affect loops that are highly flexible/deformable. The β-sheets, the core of the *Calf-1* structure, maintain their conformational stability and do not undergo any change in terms of PB distributions.

Integrin structure is made of a succession of well-described structural domains, which can be easily cut by protein peeling approach [39,40,41]. Hence, different combinations of domains from integrin α_IIb_β_3_ have been tested, namely *Calf-1*, *Calf-1* + *Calf-2* and *Thigh* + *Calf-1*. Crystal coordinates for *Calf-2* domain have missing regions located in the large connecting loops of the immunoglobulin fold. They were completed using restraints guided by biophysical expertise and then subjected to MD simulations. The dynamics of the completed loops revealed high intrinsic flexibility, but not any disorder (as defined by *N*_eq_ measure [30,31,42]). It is interesting to note that some segments are more rigid than others, which underwent large amplitude of conformational changes. It also showed that the significant loop flexibility does not affect the overall stability of the *Calf-1* & *Calf-2* structure.

Located close to the connection of *Calf-1* to *Calf-2*, P741R mutation was analyzed again in this work to validate the observations from previous context (isolated *Calf-1*). MD simulations provided similar results with or without the presence of *Calf-2* domain, thus validating the possibility to use only sub-domains in the analysis of this big complex structure.

The six studied variants do not cause any major conformational modification at the sites of the mutated residues and their immediate vicinity. On the other hand, local structural differences appear in other places of the domain, as seen in our previous work [5,32]. All of the results, after comparing simulated trajectories of wild-type-to-variant systems underlined potential structural allosteric mechanisms. In most of the variants, a minimal local structural change was observed at the impact site, whereas flexibility profiles changed maximally at distant sites. A common observation is that the structural impact caused by loss of interactions at the impact site is balanced out by compensatory interactions assisted by adjustments in the connecting loops. Thus, loops act as compensatory back-up for integrins. This study was carried out with associated domains (*Calf-1* + *Calf-2* and *Thigh* + *Calf-1*) for variants of the *Thigh* and *Calf-2* domains, which correlates well with the previous results. These variants have subtle-to-no effects on the structure at the substitution sites, but induce notable allosteric effects. The variants associated with Glanzmann thrombasthenia slightly affect the local structural conformations as they are compensated by modifications of nearby molecular interactions. Moreover, the largest changes are observed at a distance from the mutated sites noted by an increase in the mobility or deformability of the most flexible regions. So, it is particularly interesting to note that, even if the RMSF of all the systems have very similar profiles, the *N*_eq_ values of some particular loops are impacted and that helped to identify long-distant changes more efficiently. It should be noted that the replicates of the different systems were analyzed independently and showed that they were particularly similar with both the RMSF and the *N*_eq_.

In conclusion, this new study shows that analyses of variants associated with pathologies of integrin α_IIb_β_3_ can be apprehended using molecular dynamics experiments. The impact of forcefields or adjacent domains is limited. Additionally, it is surprising to observe that some variants have the highest impact on adjacent domains as well, underlining the well-connected organization of the integrin’s structure. This makes them a potential candidate to study structural allostery and its mechanistic propagation.

## 4. Materials and Methods

### 4.1. Structural Data

The *Thigh* (*+knee*), *Calf-1* and *Calf-2* domains of α_IIb_ were extracted from a 2.55Å resolution crystal structure of α_IIb_β_3_ integrin (PDB code 3FCS [4]). The *Calf-1* is a domain of 141 residues (positions 603–743); it is followed by *Calf-2*, a domain of 216 residues (positions 744–959) and preceded by *Thigh* (*+knee*), a domain of 216 residues (positions 452–602). All missing atoms and missing residues were completed using Modeler software v.9.18 [43]. The GT aa substitutions were introduced in the integrin structure by in silico mutagenesis using special wizard tool in PyMOL software [20] and the SCWRL method [44]. The effects of all mutations were studied exclusively. *Calf-1* loop locations are Loop 1: 603–612, Loop 2: 620–629, Loop 3: 639–646, Loop 4: 653–57, Loop 5: 663–673, Loop 6: 678–683, Loop 7: 691–696, Loop 8: 707–715, and Loop 9: 724–735. *Calf-2* loop locations are Loop 1: 742–747, Loop 2: 755–757, Loop 3: 761–776 (completed using Modeler), Loop 4: 788–795, Loop 5: 805–812, Loop 6: 821–824, Loop 7: 829–884 (completed using expert modeling), Loop 8: 893–897, Loop 9: 909–921 and Loop 10: 931–945.

### 4.2. Studied Variants

Six different variants were studied. P741R *Calf-1* domain variant studied in this paper is involved in GT and has been analyzed in our previous study [5]; it severely impaired α_IIb_β_3_ expression (less than 5%). H798P and S926L were located in the *Calf-2* domain, whereas D560A, R250W, and S472N in the *Thigh* domain. PolyPhen-2 (Polymorphism Phenotyping v2) [34] was used to predict the possible deleterious effect on the expression/function of an amino acid substitution in the structure.

### 4.3. Molecular Dynamics

MD simulations were conducted using GROMACS 5.1.1 software [45] with the OPLS-AA *ff* [46] and Gromos 54a7 *ff* [47], where applicable. Before starting any simulation experiments, each structural variant was energy minimized for 500 steps of steepest descent and 500 steps of conjugate gradient optimized by SHAKE algorithm, using GROMACS suite. WT and variant forms of *Calf-1* alone, *Calf-1 + Calf-2* and *Thigh + Calf-1* were soaked in a rhombic dodecahedral simulation box with TIP3P water molecules and neutralized with Cl^−^ ions. The MD protocol was standardized through our previous works [5,33]. After 1 nanosecond (nsec) of equilibration (with position restraints on the protein), each system was simulated through 11 independent production runs for a total of 850 nanoseconds as in [5]. Molecular conformations were saved every 100 picosecond (psec) for downstream analysis. The first 5 nsec of each MD simulation were discarded, as the residues at the extremities could not be taken into calculations. Trajectory analyses were conducted with the GROMACS software, in-house Python and R scripts. Root mean square deviations (RMSD) and root mean square fluctuations (RMSF) were calculated on Cα atoms only. Residue interactions were analyzed using the online tool PIC (protein interactions calculator) [48]. A total of 11 new different systems were tested for this study (see Figure 2 for summary of the 11 different systems simulated).

### 4.4. Protein Block Analysis

Protein blocks (PBs) are a structural alphabet composed of 16 local prototypes [42]. Each specific PB is characterized by the φ, ψ dihedral angles of five consecutive residues with each PB assignment focused on the central residue. Obtained through an unsupervised training approach and performed on a representative non-redundant databank, PBs give a reasonable approximation of all local protein 3D structures [49]. PBs are very efficient in tasks, such as protein superimpositions [50] and MD analyses [32]. They are labeled from *a* to *p*; PBs *m* and *d* can be roughly described as prototypes for α-helix and central β-strand, respectively. PBs *a* to *c* primarily represent β-strand N-caps and PBs *e* and *f* representing β-strand C-caps; PBs *a* to *j* are specific to coils; PBs *k* and *l* to α-helix N-caps whereas PBs *n* to *p* to α-helix C-caps. The PB assignment was carried out using our PBxplore tool (available at GitHub) [29].

PB assignments were conducted for each residue of the *Calf-1* domain and over every snapshot extracted from MD simulations. The equivalent number of PBs (*N*_eq_) is a statistical measurement similar to entropy that represents the average number of PBs for a residue at a given position. *N*_eq_ is calculated as follows [42]:

(1) $$N_{\mathrm{eq}}=\exp(-\sum_{x=1}^{16}f_{x}\ln f_{x})$$

where *f_x_* is the probability of PB *x*. A *N*_eq_ value of 1 indicates that only 1 type of PB is observed, whereas a value of 16 is equivalent to a random distribution. To underline the main differences between the wild-type (WT) and a variant for each position, a Δ*N*_eq_ value is computed. Δ*N*_eq_ is the absolute difference between corresponding *N*_eqs_.

However, a same Δ*N*_eq_ value can be obtained with different types of blocks in similar proportions. To detect a change in PB profile, a ΔPB value was calculated. It corresponds to the absolute sum of the differences for each PB between the probabilities of a PB *x* to be present in the WT and the variant forms (*x* goes from PB *a* to PB *p*). ΔPB is calculated as follows [5]:

(2) $$\Delta \mathrm{PB}=\sum_{x=1}^{16}|(f_{x}^{\mathrm{WT}}-f_{x}^{\mathrm{var}})|$$

where $f_{x}^{\mathrm{WT}}$ and $f_{x}^{\mathrm{var}}$ are the percentages of occurrence of a PB *x* in, respectively, the WT and the variant structures. A value of 0 indicates perfect PBs identity between WT and variant, whereas a score of 2 indicates a total difference.

## Data Availability

Starting structure from our study is available on the PDB website: https://www.rcsb.org.

## Supplementary Materials

The following supporting information can be downloaded at https://www.mdpi.com/article/10.3390/ijms23020858/s1. References [51,52,53,54,55,56,57,58] are cited in the Supplementary Materials.

## Abbreviations

Fetal/Neonatal Alloimmune Thrombocytopenia (FNAIT), Glanzmann thrombasthenia (GT), Human Platelet Alloantigens (HPA), Molecular Dynamics (MD), Number of equivalent (*N*_eq_), Proteins Blocks (PBs), Root Mean Square Deviation (RMSD), Root Mean Square Fluctuation (RMSF), wild-type (WT).
