# Supplementary material for: Analysis of Integrin αIIb Subunit Dynamics Reveals Long-Range Effects of Missense Mutations on Calf Domains
Source: Int J Mol Sci. 2022 Jan 13;23(2):858. doi: 10.3390/ijms23020858 (PMC8776176; doi:10.3390/ijms23020858)
Supplement: Supplementary file 1 [file ijms-23-00858-s001.zip › ijms-1509076-Supplementary-Material-Modified-Version-FINAL.pdf]

Supplementary Material

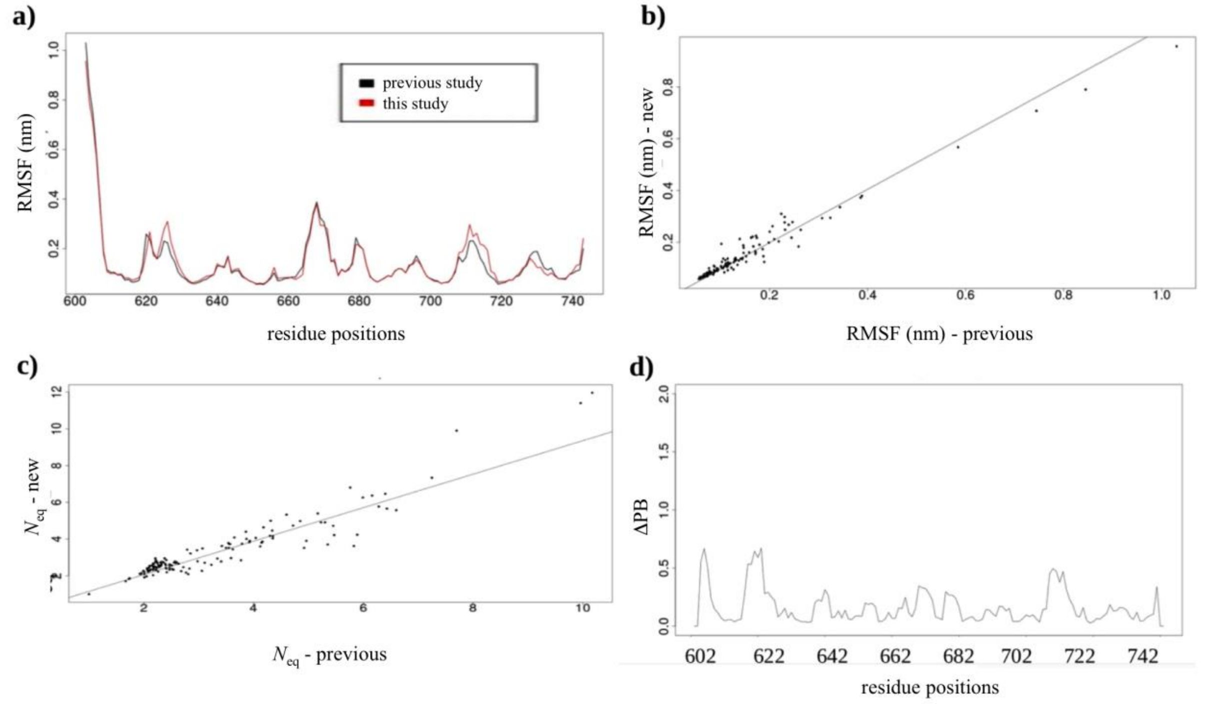

Figure S1. Comparison of simulation of Calf-1 domain. (**a**) RMSF values of each residue between previously generated data (in black) and new one (in red). (**b**) RMSF correlation. (**c**) N_{eq} correlation. (**d**) ΔPB between previous and new generated data.

The first analysis was simply to test the robustness of Gromacs evolution on *Calf-1* domain as done in [5]. The whole system is made up of 22,724 atoms, 11 independent dynamics were performed for a cumulative duration of 850 ns simulation for the wild system (WT). The simulations carried out were analysed for the evolution of temperature, pressure and energy values (potential, kinetics and total). They all show satisfactory values, similar to those of the previous study [5].

Figure S1a shows the superposition of RMSF values obtained in the previous study and this one. Figure S1b highlights their correlation as well as the Figure S1c for N_{eq} values, both shows excellent correlation values of 0.98 and 0.94, respectively demonstrating the excellent comparability of the experiments. As expected, the Figure S1d which presents the values of ΔPB of the WT between these simulations and shows slight differences between the two works ranging between of 0 to 0.6 with a median value of 0.15 underlying excellent compatibility. Residues 620–621 & 625–626 (loop 2), 667–670 (loop 5) and 711–713 (loop 8) have small amplitudes but highlight the same mobile regions as those identified in the previous study (see Figure 5 of [5]).

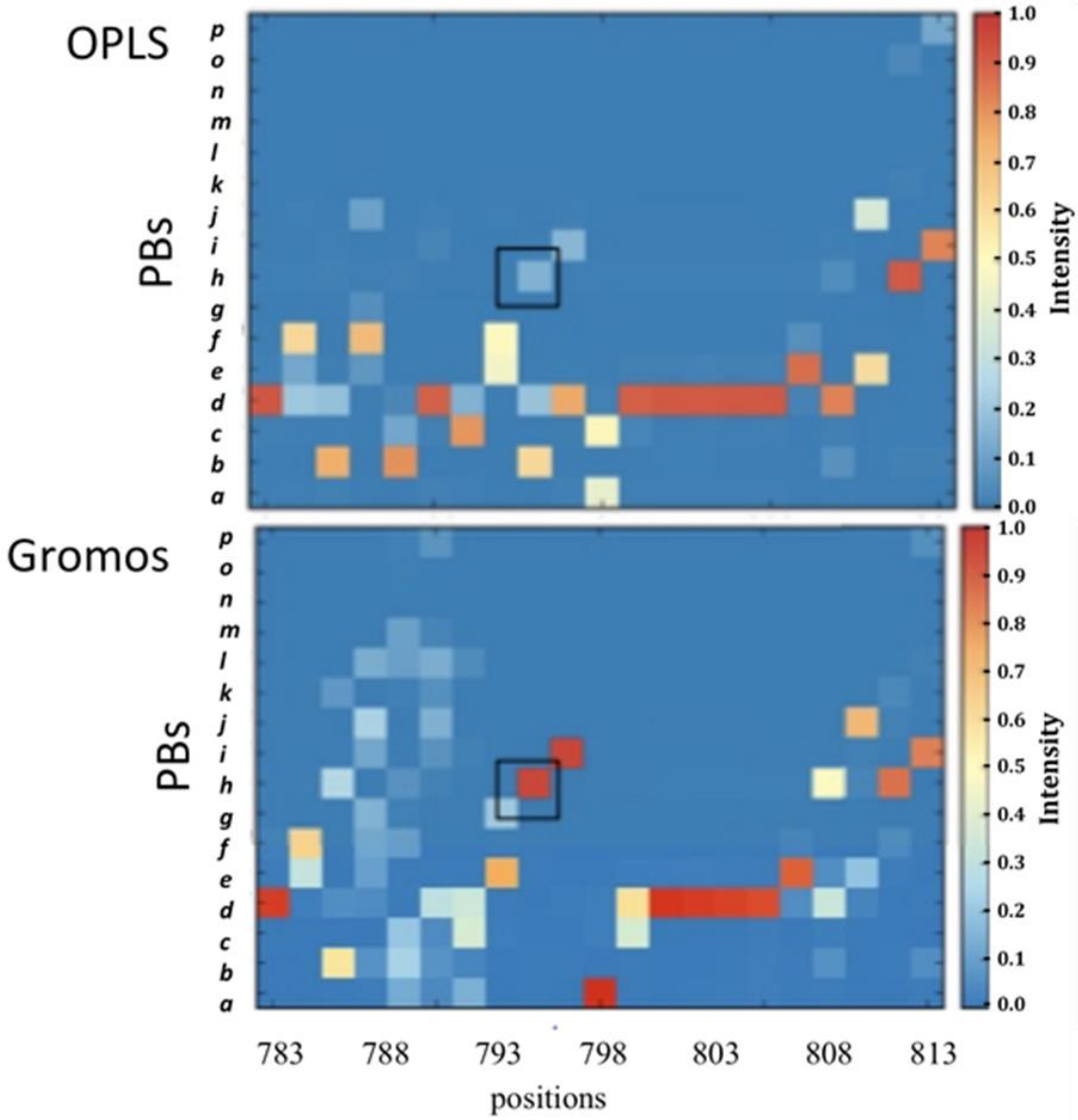

Figure S2. OPLS ff vs Gromos ff. PBs occurrence map [29] of positions 783 to 813 for OPLS *ff*, and (2) Gromos *ff*.

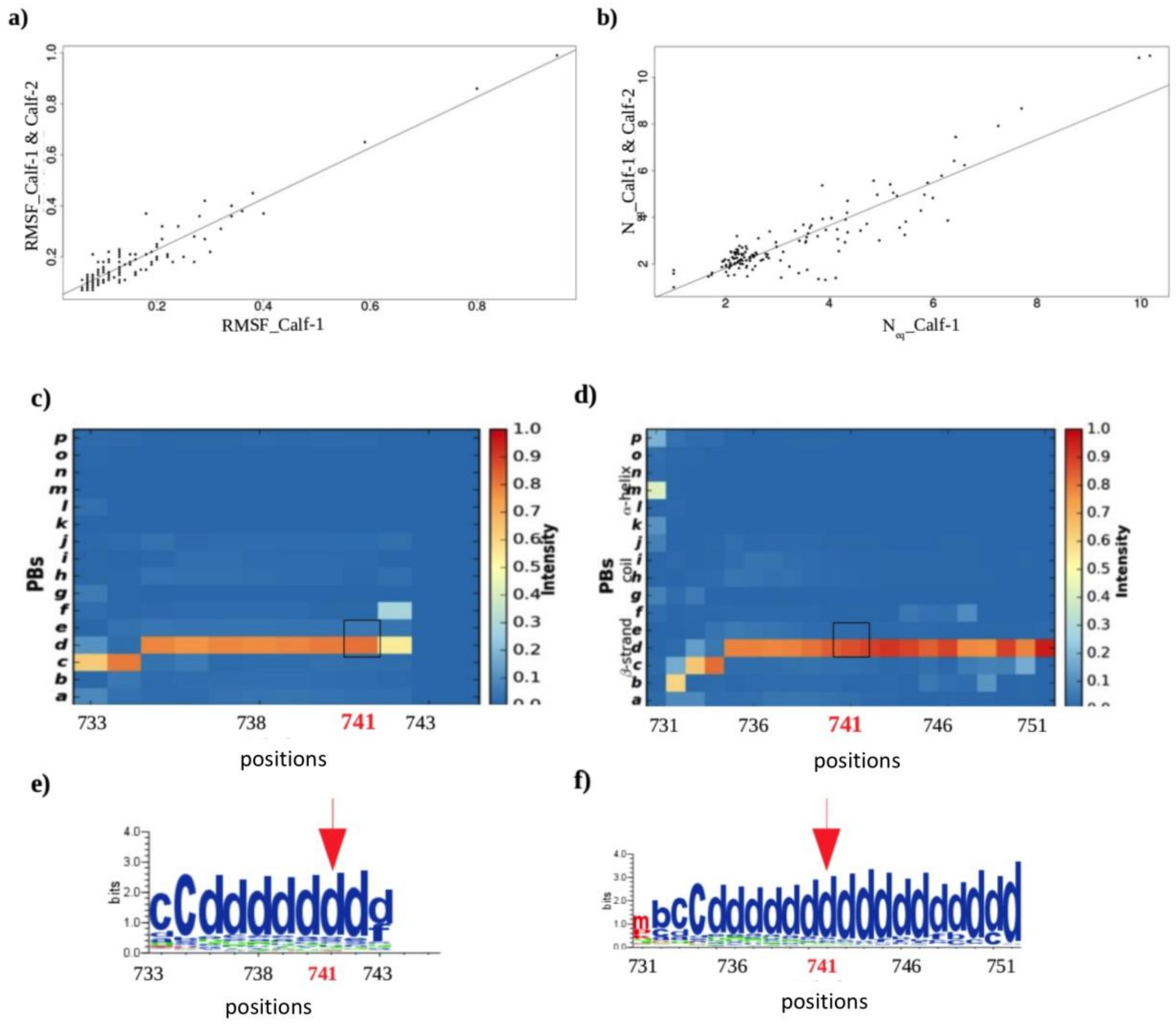

Figure S3. P741 of Calf-1 domain. (**a**) RMSF and (**b**) N_{eq} for the variant of *Calf-1* alone against *Calf-1 & Calf-2*. Occurrence (**c**) of *Calf-1* alone and (**d**) for the *Calf-1* + *Calf-2* system. Logo maps (**e**) for *Calf-1* alone and (**f**) for the *Calf-1* + *Calf-2* system.

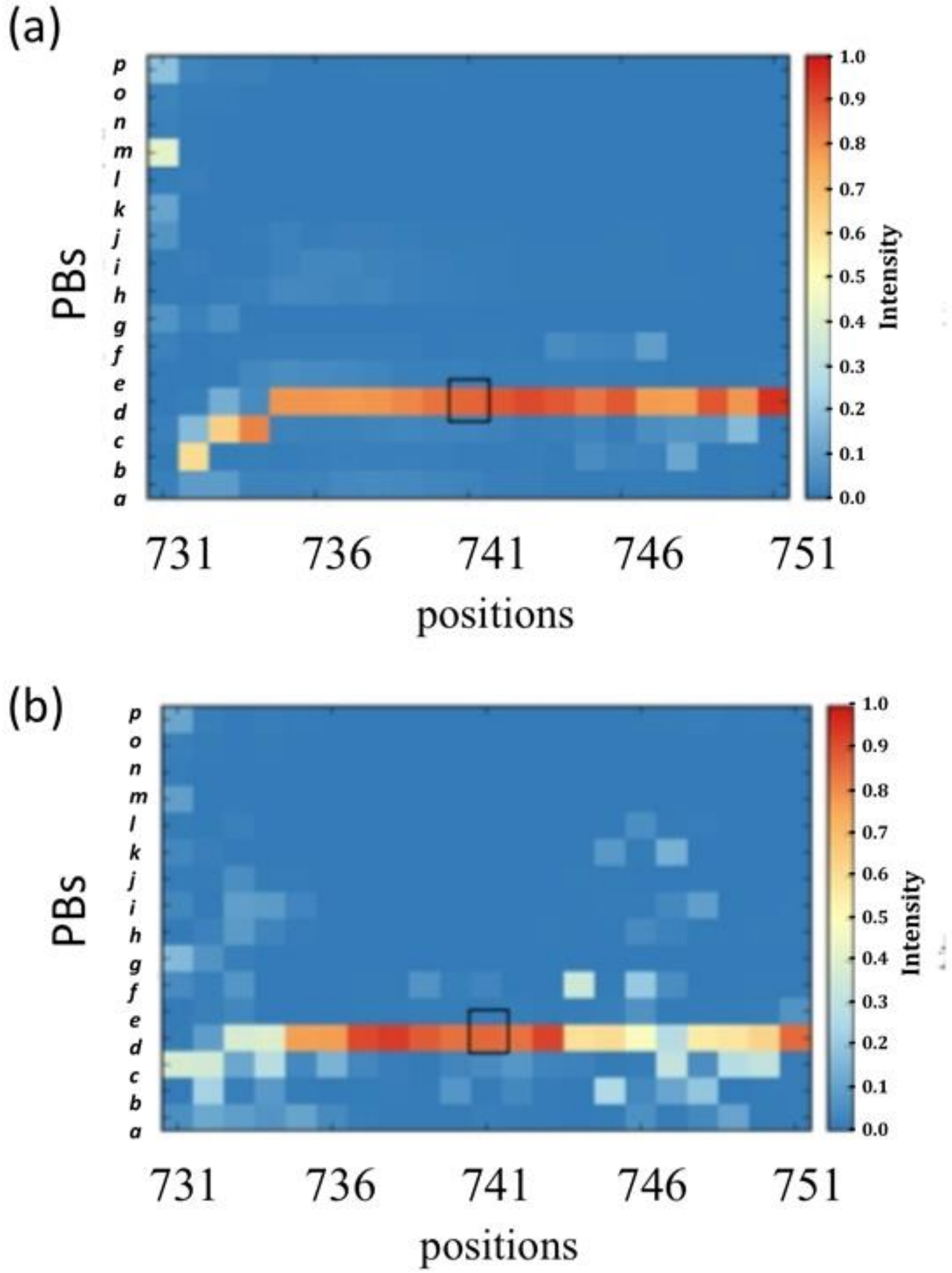

Figure S4. P741R variant. (**a**) and (**b**) depicts the PB maps for residue 741 of the wild-type and the variant systems respectively.

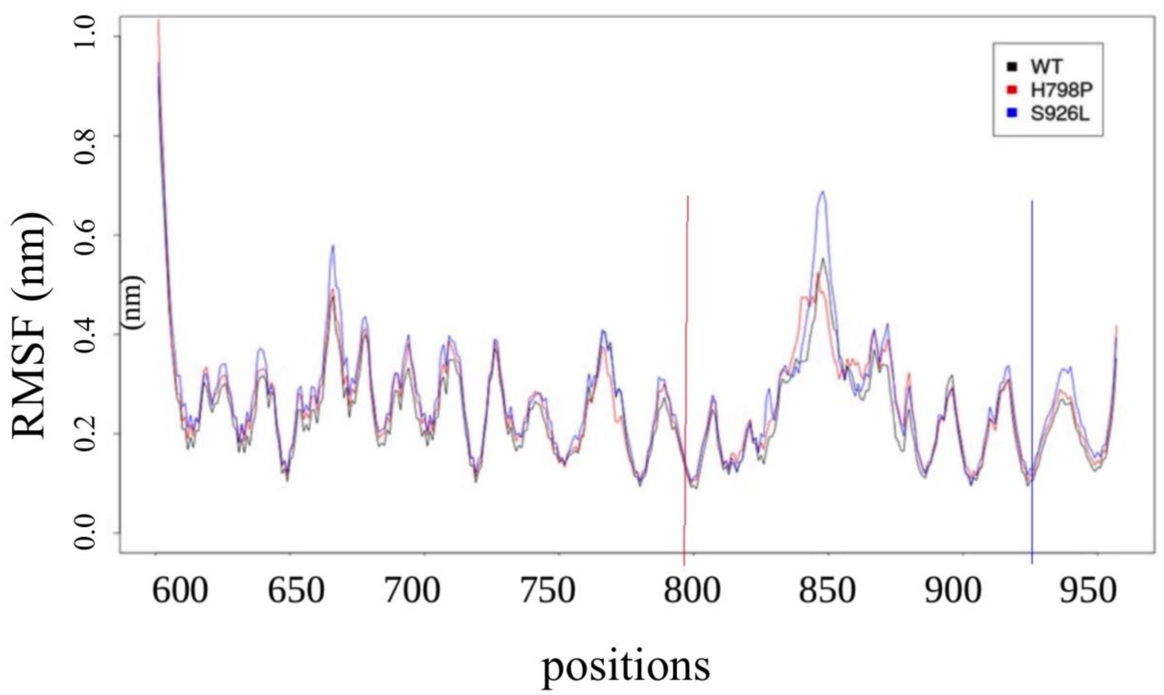

Figure S5. Calf-2 variants. RMSF values for the wild-type (in black), P798 (in red) and L926 (in blue).

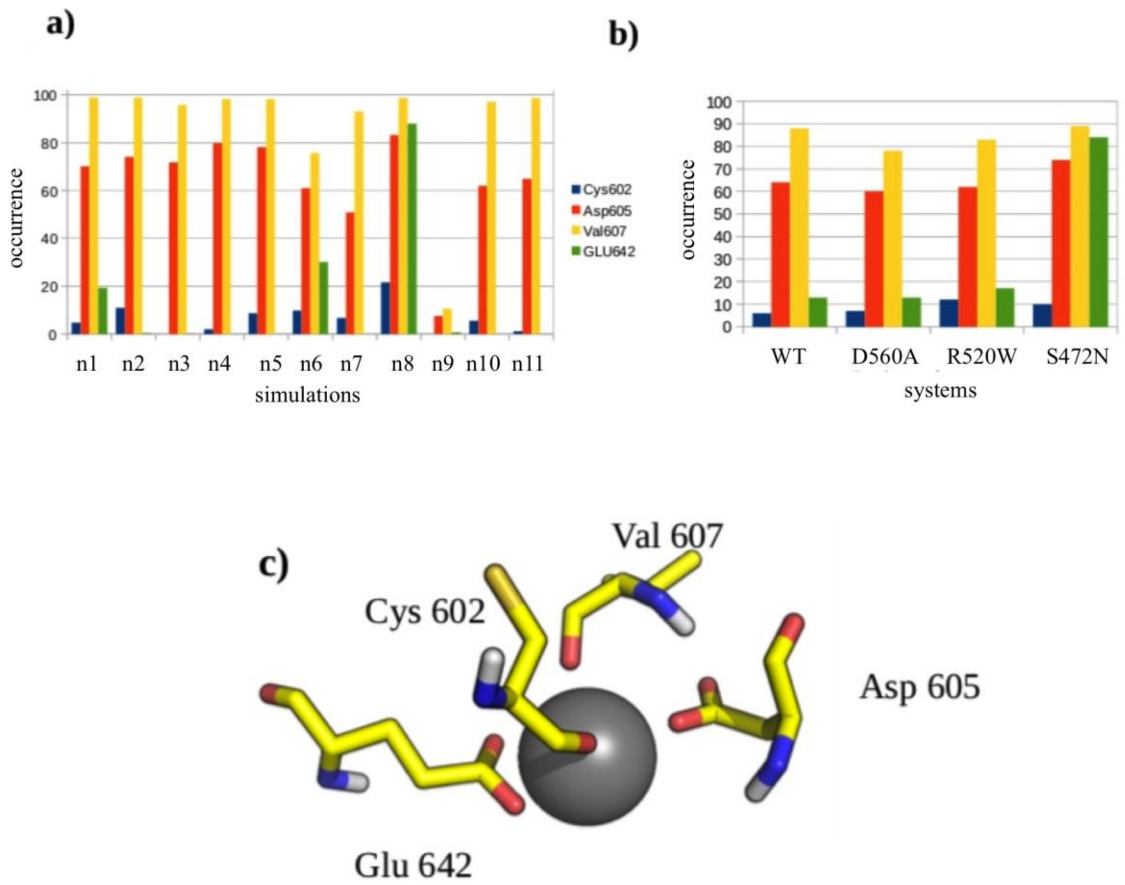

Figure S6. Ca^{2+} in Thigh domain. Ca^{2+} occurrence at the binding site during the dynamics (**a**) for the 11 simulations of the wild-type sequence and (**b**) average for the four systems. (**c**) Visualisation of the four interactions with Ca^{2+}.

These results are consistent with another study, which shows that the affinity of Ca^{2+} can vary during a molecular dynamics simulation [38]. This last study focuses on ◎-parvalbumin. A simulation on a wild-type system and also three variants were performed. They show that the affinity of Ca^{2+} varies during this dynamic. As these are weak interactions, it is therefore not surprising to observe link breaks during the simulations.

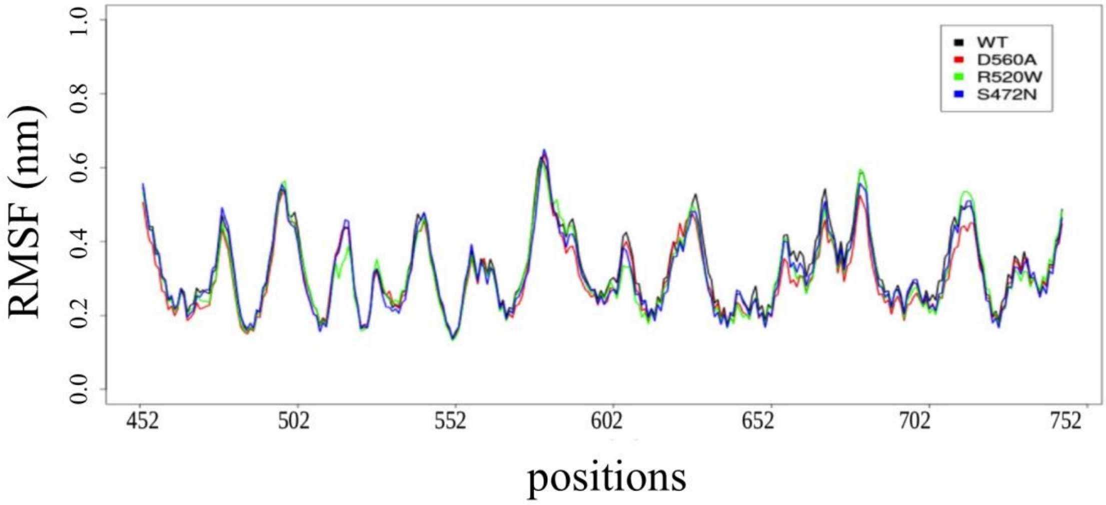

Figure S7. Thigh variants. RMSF values for the wilt-type (in back), A560 (in red), W520 (in green) and N472 (in blue).

Table S1. Thigh + Calf-1 simulations. (**a**) mean ΔN_{eq} and interactions, (**b**) ΔNeq and ΔPBs, (**c**) PB profiles for *Thigh - Calf-1* domains with wild type (D560, R520 and S472), and variants for the three systems (A650, W520 and N472).

			**Interactions**							
	**Mean ΔN_{eq}**		**Wild-Type**				**Variants**			
	Thigh	*Calf-1*	hydrogen	ionic	hydrophobic	aromatic	hydrogen	ionic	hydrophobic	aromatic
D560A	0.45	0.56	4	1	/	/	4	/	/	/
R520W	0.42	0.48	5	2	/	/	2	/	4	1
S472N	0.40	0.25	1	0	/	/	1	/	/	/

	**Mean ΔN_{eq}**	
	Thigh	*Calf-1*
D560A	0.45	0.56
R520W	0.42	0.48
S472N	0.40	0.25

	**Wild-Type**																**Variants**															
PBs	a	b	c	d	e	f	g	h	i	j	k	l	m	n	o	p	a	b	c	d	e	f	g	h	i	j	k	l	m	n	o	p
D560A		0.3	0.1													0.6		0.2	0.1													0.7
R520W	0.2					0.5							0.3				0.1					0.4							0.5			
S472N		0.3						0.1				0.6						0.3										0.7				

Results: Structural Modelling of the Calf-1 and Calf-2 Domains

1. Structural Data

In this study, the *Calf-2* domain (residues 744 to 952) has to be completed as structural information for two (2) regions is missing in the selected template (unresolved in the crystallographic structure). The first region is 11 residues long (positions 763–775) whereas the second one is 34 residues (positions 840–873). The second region is long enough to be beyond the scope of default loop-modelling algorithms [21]. The secondary structure prediction by PsiPred [22] and Jpred [23] did not identify any repetitive structures in the region, whereas PredyFlexy showed high flexibility content [24–26]. To complete these missing regions, homologous sequences with solved structures in Protein Databank (PDB) were searched using PSI-BLAST [27]. Five (5) structures were identified belonging to the alpha subunit of α_v family and are all very similar to each other. Two of these structures (PDB codes: 4G1E [51] and 3IJE [52]) have percentage sequence identity of 38% to the *Calf-2* domain and covers 91% of the domain. Additionally, they have resolutions of 3.0 Å and 2.9 Å respectively. From structural modelling perspective, both of them have the coordinates for the missing loop one but 4G1E covers the second large loop better (17 residues missing) than 3IJE (28 residues missing). Its *Calf-2* domain is also - in terms of fold – highly similar to 3FCS (RMSD value of 0.72 Å and TM-score [83] of 0.95), which is the selected template for *Calf-1* domain modelling. Thus, 4G1E was selected as the additional structural template to complete *Calf-2* domain where 3FCS coordinates would cover the overall topology and 4G1E is aligned at the missing loop regions. The multi-template modelling was performed using Modeller Software [43]. A total of 100 models were generated and final selection was made using DOPE scores (Discrete Optimization of Potential Energies) [54]. However, the large missing loop could not be properly modelled in any of the best models selected, as all the models were highly compact, i.e., at the opposite of what must have been achieved.

2. Expert-Guided Loop Modeling in Calf-2 Domain

In order to find structural coordinates for the missing loop, a second strategy was used by searching for structurally similar entries in the PDB regardless of their homology to $\alpha_{IIb}\beta_3$. FATCAT algorithm (Flexible structure AlignmenT by Chaining Aligned fragment pairs allowing Twists) [28] was ran on the structural database, PDB to find similar structures. This structural comparison approach allows rigid body movement in order to align structures in a flexible manner; it was further confirmed using iPBA approach [55–57]. The resulting match, (PDB code: 4NEH [58], resolution: 2.75 Å), has a sequence identity of only 24%, but the fold is similar as it belongs to $\circledcirc_x$ integrin subunit. In 4NEH, the *Calf-2* domain is shorter and consists of only 190 residues but with no missing residues.

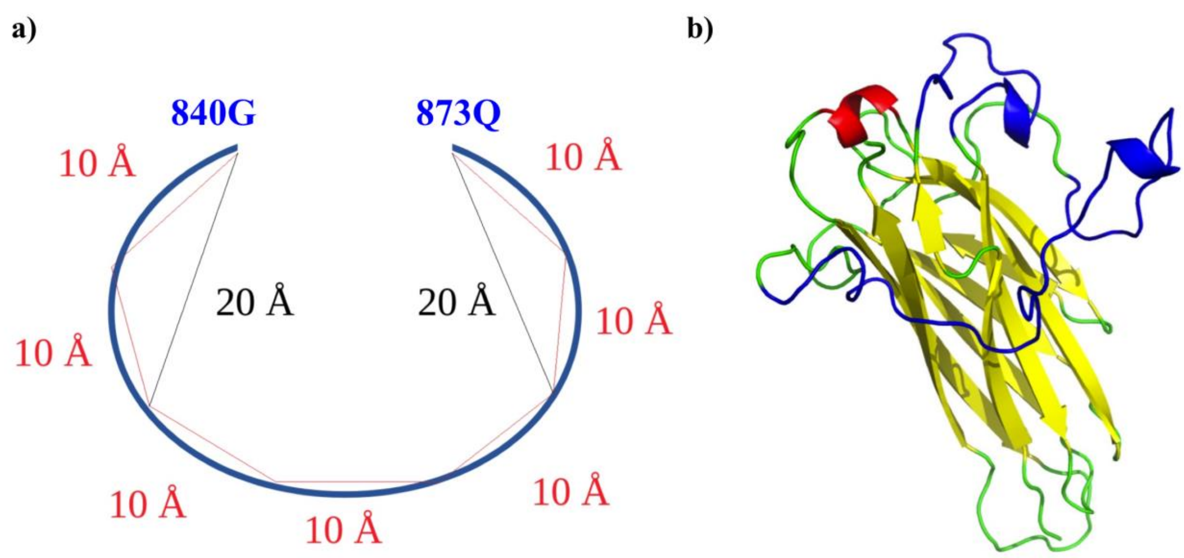

Figure S8. *Calf-2* missing regions. (a) Set of constraints imposed on the second missing region of *Calf-2* (positions 840G - 873Q). (b) Completed model of *Calf-2*, shown in PyMOL [20]. The ◎-strands are colored in yellow, the ◎-helices in red and the loops in green. The two missing regions are colored in blue.

The completion of the missing loops in 3FCS was performed using Modeller software [43], by aligning the 3FCS sequences to available coordinates in the corresponding regions of to the other two integrins (4G1E and 4NEH). One hundred structural models were generated; the model with the lowest DOPE score was retained. Different optimization attempts (loops...) have been tested. However, the attempts failed each time as the second loop was found to be deformed and folded back onto to the IgG fold of the *Calf-2* domain. Such folding could not be biologically true, therefore, to better model this missing region we employed the basics of protein biophysics and devised distance and angular restraints after assessing the loop geometry in other immunoglobin folds. The distance constraints had to be imposed to keep the modelled loop away from structure, avoiding unnatural interactions between the loop and the *Calf-2* domain. Figure S8a shows the principle implemented with distance restraints between 5 consecutive residues of 10 Å and two distance restraints of 20 Å (between the first 840G residue of the loop and the 850H, likewise between the last residue 873Q and residue 863L). Those choices were made as they are needed to ensure that loops were not directly interacting with the rest of the structure (a behaviour that is missed by MODELLER's algorithm). All the generated models were inspected manually and finally the model that was least impacted by completion, had a low Root Mean Square Deviation (RMSD) to the template and with correct loop geometry was selected. This model (see Figure S8b) which has an excellent RMSD value of 0.2 Å with X-ray structure.
